# Process evaluation of a tailored work-related support intervention for patients diagnosed with gastrointestinal cancer

**DOI:** 10.1007/s11764-019-00797-3

**Published:** 2019-11-19

**Authors:** AnneClaire G. N. M. Zaman, Kristien M. A. J. Tytgat, Jean H. G. Klinkenbijl, Angela G. E. M. de Boer, Monique H. W. Frings-Dresen

**Affiliations:** 1 Amsterdam UMC, Coronel Institute of Occupational Health, Amsterdam Public Health Research Institute, University of Amsterdam, Amsterdam, The Netherlands; 2 Amsterdam UMC, Department of Gastroenterology, University of Amsterdam, Amsterdam, The Netherlands; 3 Department of Surgery, Gelre Hospitals, Apeldoorn, the Netherlands; 4 University of Amsterdam, Amsterdam, the Netherlands

**Keywords:** Return to work, Gastrointestinal neoplasms, Vocational rehabilitation, Patient care, Process evaluation

## Abstract

**Purpose:**

To perform a process evaluation of a tailored work-related support intervention for patients diagnosed with gastrointestinal cancer.

**Methods:**

The intervention comprised three tailored psychosocial work-related support meetings. To outline the process evaluation of this intervention, we used six key components: recruitment, context, reach, dose delivered, dose received and fidelity. Data were collected using questionnaires, checklists and research logbooks and were analysed both quantitatively and qualitatively.

**Results:**

In total, 16 hospitals, 33 nurses and 7 oncological occupational physicians (OOPs) participated. Analysis of the six key components revealed that the inclusion rate of eligible patients was 47%. Thirty-eight intervention patients were included: 35 actually had a first meeting, 32 had a second and 17 had a third. For 31 patients (89%), the first meeting was face to face, as per protocol. However, in only 32% of the cases referred to support type A (oncological nurse) and 13% of the cases referred to support type B (OOP), the first meeting was before the start of the treatment, as per protocol. The average duration of the support type A meetings was around the pre-established 30 min; for the OOPs, the average was 50 min. Protocol was easy to follow according to the healthcare professionals. Overall, the patients considered the intervention useful.

**Conclusions:**

This study has shown that the strategy of tailored work-related support is appreciated by both patients and healthcare professionals and applicable in clinical practice.

**Implications for Cancer survivors:**

The intervention was appreciated by patients; however, whether the timing of the work-related support was adequate (i.e. before treatment was started) requires further research.

**Trial registration:**

NTR5022.

**Electronic supplementary material:**

The online version of this article (10.1007/s11764-019-00797-3) contains supplementary material, which is available to authorized users.

## Introduction

The number of working-age people (i.e. people aged 18–67 years) diagnosed with cancer is growing [1–3]. As a result of this, and knowing that a large number of these people experience work-related problems such as fatigue [4, 5] and cognitive problems [6], it is important to provide work-related support. Furthermore, the diagnosis of cancer is associated with an increased risk for sick leave or even disability pension [7, 8].

In recent years, we have seen the emergence of an increasing focus on the aspects of psychosocial support for patients diagnosed with cancer [9], including return to work (RTW) [10].

Work-related support interventions are available and include e.g. psychosocial patient counselling and education with or without physical training components [11, 12]. However, (work-related) problems differ in the severity per person and therefore, a tailored approach is preferred. Many intervention studies are addressed to patients with, for example, breast cancer [11, 13, 14]. We focussed on patients diagnosed with gastrointestinal (GI) cancer, which include malignancies of the digestive track system and are relatively prevalent for both men and woman with an incidence in the top 15 of cancer types [15, 16]. Considering that only a limited number of RTW intervention studies [11] include GI malignancies, we developed a tailored psychosocial work-related support intervention [17] named GIRONA (Gastro Intestinal cancer patients Receiving Occupational support Near and After diagnosis). Our intervention includes three individual 30-min face-to-face meetings, with the first scheduled prior to the start of the treatment, as it is known from previous research that work-related problems can occur at the moment of diagnosis [18]. The aim of the intervention was to inform patients about work during and after treatment and to identify any existing work-related problems. Patients were informed about, for example, the importance of work and asked whether they already had contact with the work environment and whether their employer/colleagues knew about their diagnosis.

Since work-related problems can differ in severity, the intervention itself was split into three types of work-related support (support types A, B and C) in order to meet the needs of individual patients. They were referred to a particular type, based on certain possible contributing factors to work-related problems. These were described in a decision diagram [17], for example, fatigue and/or uncertainty about the future in relation to work which will be discussed in support A and lack of support from colleagues and employer and/or cognitive problems in relation to work in support B. Furthermore, for each support type, the kind of healthcare professional assigned to provide supportive care was tailored to the severity of the work-related problems. For support type A, this was an oncology nurse, for type B, an OOP and for type C, a multidisciplinary team (which included at least an oncology nurse, the treating physician and an OOP).

It is important to know whether the process of this intervention was performed as intended (i.e. per protocol). By describing the processes of an intervention, the detailed information required to gain and then disseminate knowledge concerning the success or failure of the execution of the process is collected [19]. It allows to understand which elements of the intervention needs more attention [20]. Therefore, the results from this process evaluation can be used to understand how and why the intervention can work in clinical practice, as well as to improve work-related support interventions, including early interventions, in general [19, 21, 22]. A process evaluation addresses different elements (i.e. recruitment, context, reach, dose delivered). To structure and describe those, we used the model of Linnan and Steckler [21] for structuring the process evaluation. Other RTW studies [23–25] also evaluated their intervention process with this model, allowing us to relate and learn from their intervention process. Furthermore, results of the effectiveness of an intervention can be put into perspective by performing a process evaluation with the individual components of the intervention process and the relation between these components [20, 26].

In summary, the objective of this study was to perform a process evaluation of our developed tailored work-related support intervention for patients diagnosed with gastrointestinal (GI) cancer.

## Methods

### Study design

This process evaluation was part of the GIRONA study (Gastro Intestinal cancer patients Receiving Occupational support Near and After diagnosis), which included a multicentre Randomised Controlled Trial (RCT). The full study design of the RCT was published previously [27].

For the process evaluation, we collected qualitative and quantitative data on the process indicators using (1) GIRONA study follow-up questionnaires completed by the patients, (2) checklists completed by the healthcare professionals during the intervention meetings, (3) research logbooks completed by the research team during the GIRONA study and (4) a questionnaire on the substantive process outcomes after the intervention was performed/received, completed by both the patients and the healthcare professionals.

### Participants

Patients in the GIRONA study were diagnosed with GI cancer to be treated with curative intent, aged 18–63 years, had paid work at time of diagnosis and were on sick leave. The healthcare professionals who participated in the GIRONA study included oncological GI nurses from the participating hospitals and oncological occupational physicians (OOP). These OOPs had undergone an accredited, specialised training programme that included a theoretical module and a traineeship, which prepared them to support patients diagnosed with cancer. These OOPs were not affiliated to the hospital, in which the patient received treatment, nor to the patient’s own workplace.

### The GIRONA intervention procedure

Prior to the start of the GIRONA study, the oncology nurses attended a training session, during which they learned about the study’s objectives and activities, certain processes of good clinical practice research (e.g. the informed consent procedure), the problems faced by patients diagnosed with cancer regarding work/RTW and basic knowledge about the Improved Gatekeeper Act, which takes effect when an employee/patient is granted sick leave. The training session lasted about 2–2.5 h; it was given by the researcher [AZ] at the participating hospitals.

The OOPs were informed about the GIRONA study’s objectives and activities.

Patients were asked to participate at the hospital where they were receiving treatment. The oncologist or oncological nurse checked whether the patient was eligible for this study during the first visit to the hospital. A brief explanation was given and the patient was asked whether one of the researchers of the project team could contact them by telephone.

Patients who agreed to participate signed an informed consent form and subsequently received the baseline questionnaire. When the questionnaire was returned, randomisation took place and patients were informed about the outcome of the randomisation process. If they were randomly allocated to the intervention group, the decision diagram was filled out by the one of the researchers of the project team, based on the data from the baseline questionnaire. The healthcare professional was informed so she/he could make an appointment for the first meeting.

### Process evaluation components

To outline the process evaluation, we adapted the model of Linnan and Steckler [21] and used the following key process evaluation components: recruitment, context, reach, dose delivered, dose received and fidelity.

#### Recruitment

Recruitment encompassed the procedures used to approach the potential participants [21]. A description was given of the recruitment strategy for hospitals and patients. We measured the proportion of hospitals that participated compared with the number that had been invited to participate. In this process evaluation component, patients who participated in the study were asked whether they were satisfied with the timing of the invitation to participate in the study.

#### Context

Context is the environment that may influence the intervention implementation [21]. To gain insight into this component, a description of the involved patients and healthcare professionals was presented. In each participating hospital, at least one oncological nurse was proposed to perform the intervention. Contact was established with the nurses through the local investigators (mostly medical doctors). Each participating hospital was covered by an OOP. The OOPs were selected based on the travel distance from their facility to the participating hospital. The baseline questionnaire was used to collect patient information, and the researcher’s logbooks of the project team were used to collect descriptions of the hospital and healthcare professional.

#### Reach

Reach is the intended target proportion of participants, represented as the attendance rate [21]. This was defined as the proportion of patients who participated compared with all eligible patients. When patients were willing to give a reason for non-participation, the reason was recorded.

#### Dose delivered

Dose delivered encompassed the actual and intended proportion of intervention delivered per protocol [21]. This was measured as the proportion that was delivered to the patients from the start of the intervention and that was actually delivered compared with the intervention protocol. The protocol prescribed three meetings: the first was face to face and given before the treatment was started; the second and third meetings could be conducted face to face or by phone. After the second meeting, the healthcare professional filled out the decision diagram to check that the support was appropriate and whether a third meeting was needed. All meetings were scheduled to last 30 min. The intensity of these intervention components per patient was measured by the number of meetings actually held, the number of patients referred to each type of support, the number of face-to-face meetings, the duration of the meetings and whether the patient was referred to another type of support as a result of the decision diagram. These data were registered by the healthcare professionals and in the researcher’s database of the project team. The intervention was completed according to protocol if at least two meetings took place (unless a patient indicated that no support was needed). Dose delivered was also operationalised by whether the healthcare professional discussed all items on the topic list (ESM Appendix 1).

#### Dose received

Dose received encompassed the elements of the intervention actually received [21]. This was operationalised at two levels, namely (a) patients who received the work-related support and (b) healthcare professionals.

- Patients who received the work-related support were asked about the following: the number of meetings (exactly right/not enough), the duration of the meetings (exactly right/too short/too long/not applicable), the timing of the meetings (exactly right/too early/too late/not applicable), the items discussed (good/okay/bad), whether they considered the intervention useful (yes/reasonably useful/neutral/not really/no) and their overall satisfaction (1 = very dissatisfied, 5 = very satisfied).
- Healthcare professionals: nurses who had attended a training session prior to the start of the study. We asked them about the following: the training (good/sufficient/bad), if they felt they could support patients with work-related problems (I did not need training for this/yes, I have enough knowledge/ no), the timing of the training (just right/too early/too late) and whether the training lacked any elements (yes/no).

Both nurses and OOPs were asked about the following: their role in supporting patients with work-related problems (1 = very dissatisfied, 5 = very satisfied), how they felt in their role (good/neutral/insecure/bad), whether they felt that patients know the importance of work at the moment of diagnosis (yes/no), the number of meetings (just right/too few/too many), the duration of the meetings (just right/too short/too long) and the timing of the meetings (just right/too early/too late).

#### Fidelity

Fidelity is the extent to which the intervention was performed as planned [21]. This component was measured by assessing the performance of the healthcare professionals based on the items described in the study protocol, referred as performance indicators (a–i):

- First meeting: before treatment started
- First meeting: face to face
- First meeting: 30-min duration
- Second meeting: within 6 months after the first meeting (permitted to be held by phone)
- Second meeting: 30-min duration
- Healthcare professional filled out the decision diagram after the second meeting
- Third meeting (when needed): within 9 months after the first meeting (permitted to be held by phone)
- Third meeting: 30-min duration
- Healthcare professional completed the form after each meeting.

The nine performance indicators were individually scored as yes/not applicable (score 1) or no (score 0), separately for the healthcare professionals (nurses and OOPs). A ‘yes’ was scored if the indicator was 75% or higher. For the duration of the meetings, a margin of 5 min around the average (30 min) was scored as 1. All individual performance indicator scores were weighted equally and were converted into an overall value of the nine performance indicators together. When the overall performance indicator was 75% (7 points), the intervention was performed and scored as sufficient.

**Fig. 1:**
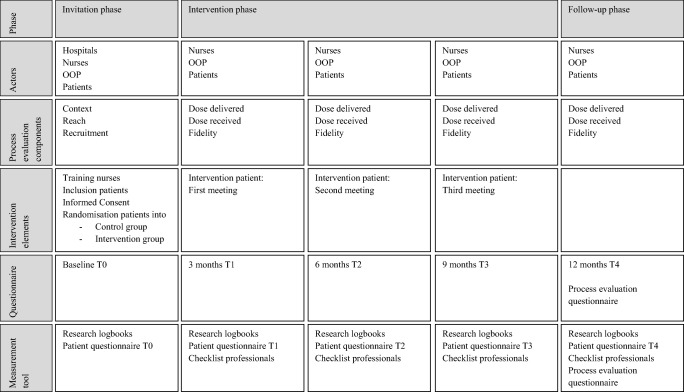
Study phases, the actors per phase, process evaluation components, intervention elements and questionnaires at different time points of measurement including measurement tool

In Fig. 1, a clear description is presented of the intervention components and process evaluation components, as suggested by Perera et al. [28] and Bakker et al. [20].

### Analysis

The data on content (recruitment and context) were analysed with qualitative content analysis using MAXQDA (VERBI GmbH, Germany). Based on the content, the patients’ answers about the timing of introducing the study were organised and analysed. Only the data on key process evaluation component ‘hospital recruitment’ were described based on the information that one of the researchers of the project team collected during contact (face to face, telephone and/or email) with the potential local investigators*.*

The data on reach, dose delivered, dose received and fidelity were described using SPSS (IBM 24.0 statistics) descriptives, namely numbers and percentages supplemented with mean and standard deviation, if appropriate.

## Results

### Recruitment

Twenty-five Dutch hospitals were contacted by email and phone, followed by a face-to-face meeting to explain the study and discuss participation. Ten hospitals declined to participate. Reasons given for non-participation included ‘limited time’, ‘no nurses/research nurses available’, ‘shortage of medical staff’ and ‘interference with other ongoing research’. The patient inclusion period was extended twice, in agreement with the local investigators. Because the date of onset varied between the hospitals, the extension time also varied between the hospitals. Patients’ reactions to the timing of their introduction to the study (i.e. at the moment they were diagnosed) were of qualitative character and ranged from ‘At such a moment any help is welcome’ to ‘Too early, as you are more inclined to participate in anything because you are very uncertain about everything at that moment’.

### Context

In total, 15 Dutch hospitals (one of which has two sites) participated, located in North and South Holland, Utrecht, North- Brabant, Limburg, Gelderland, Flevoland and Groningen. Academic medical centres and peripheral hospitals were included. Due to the local medical ethical approvals of the participating hospitals, not all started including patients at the same moment. Patients were included between July 2015 and May 2017. One hospital did not include any patients, which was added last to perform the RCT. No patients covered the inclusion criteria of this study during the time left of the inclusion period.

In total, 33 nurses attended a training session; of these nurses, 13 (39%) actually held work-related support meetings. All nurses who participated were specialised in gastrointestinal cancer; their job functions ranged from oncological nurse, nurse practitioner, research nurse to stoma care nurse. Seven OOPs, distributed over the 15 hospitals, participated in the study. Five (71%) of them held work-related support meetings.

Thirty-eight patients with GI cancer participated diagnosed with the following: small intestine (*N* = 1), colon (*N* = 30), rectal (*N* = 6) and anal (*N* = 1). The average age was 54 ± 8.1 years. Twenty-three of the patients (61%) were male (Table 1). At baseline, 42 patients were randomised to the intervention group. However, four of these patients were not included for further analysis. Reasons were entering palliative trajectory before intervention meeting was planned (*N* = 1) and first meeting was not planned before start of treatment (*N* = 3). These four patients did not wish to pursue the intervention meetings.

**Table 1:** Baseline characteristics of patients assigned to the GIRONA study

Patients characteristics	Patients who received the work-related support *N* = 38	
	*N*	%^**^
Sociodemographic		
Age (years)	54	SD 8.1, range 32–63
Gender (male)	23	61
Marital status		
Married/cohabiting	30	79
Single	7	18
Divorced/widowed	1	3
Main wage earner		
Yes	16	42
No, my partner is	3	8
Equal with partner	17	45
Educational attainment		
Primary/secondary education	8	21
Intermediate vocational education	16	42
Higher prof/academic education	14	37
Clinical characteristics		
Diagnosis		
Small intestine	1	3
Colon	30	79
Rectal	6	16
Anal	1	3
Work-related characteristics		
Occupational sector		
Healthcare/education	10	26
Sales	3	8
Industrial/transport/logistics	8	21
Business services	10	26
Other	7	18
Employment status		
Permanent employment	29	76
Temporary employment	2	5
Temporary agency worker	1	3
Self-employed	3	8
Other	3	8

^**^Calculated percentages may approach or exceed the total *N* and 100% due to missing values or rounding differences

### Reach

Based on the findings from two hospitals, which recorded the eligible patients for the study correctly and comprehensively during the whole study period, we used this information to describe the reach.

In these two hospitals, we included a total of 18 patients. Sixteen other eligible patients did not participate. Reasons for non-participation included the following: already participating in another study, treatment had already started, having no work-related problems or support for sickness absence was well organised at work. Therefore, the reach of participating patients was 47%.

The nurses were asked if they had an opinion on the fact why fewer patients were included than expected (approximately 20 patients per hospital). Some of the reasons given were as follows: ‘population screening (colorectal cancer) mainly affects patients older than 60 years’, ‘lack of time to include patients’, ‘timing of inclusion is not the moment when patients are in the process of returning to work, and patients who do not expect any problems in advance, because they have a good relationship with their employer, often reject study participation’, ‘some mistrust, afraid to burden the employer and then perhaps experience consequences of that’ and ‘patients had already arranged things well at work’.

### Dose delivered

Thirty-eight patients were included, namely 20 (53%) who had been referred to support type A and 18 (47%) who had been referred to support type B. For 35 patients (89%) (two of the 38 patients were no-shows, and for one, the meeting took place after treatment and thus was considered the second meeting), the first meeting was held face to face (Table 2). The average duration of the first meeting between patient and nurse was 30 min (range of 15–60 min). The first meetings with the OOPs lasted for an average of 55 min (range 30–90 min) (Table 3).

**Table 2:** Number of actual received meetings divided into: face to face or by phone and the number of no-shows (patients who did not show up for the meeting)

	Actual meeting received		Face to face			By phone			No-show
	*N*	%^*^	Support A	Support B	Total *N*	Support A	Support B	Total *N*	
Meeting									
1^••^	35	92	16	15^a^	31	3	1	4	2 (B)
2^•••^	32^b^	84	12^c^	6	18	6	5	11	2 (B)
3^••••^	17^d^	45	4	7	11	2	3	5	1 (B)

^*^Percentage of the total *N* = 38 intervention patients

^••^First meeting

^a^*N* = 1 no form but had face-to-face contact in support B. *N* = 1 first meeting was started after treatment and seen as meeting 2

^•••^Second meeting

^b^*N* = 1 a meeting but no information at the form about face to face or telephone (support B)

^b^*N* = 1 a meeting but no information at the form about face to face or telephone (support A)

^b^*N* = 1 no form (support A)

^c^*N* = 1 no form but had face-to-face contact (support A)

*N* = 1 meeting was cancelled due to maternity leave of oncological nurse

*N* = 3 no second meeting was needed

^••••^Third meeting

^d^*N* = 1 no form (support A)

*N* = 1 meeting was cancelled due to maternity leave oncological nurse

*N* = 19 no third meeting was needed

**Table 3:** Duration of the intervention meetings performed by the nurses and oncological occupational physicians

Meeting	Oncological nurse^*^			OOP^* (**)^		
	*N*	Minutes (SD)	Minimum-maximum minutes	*N*	Minutes (SD)	Minimum-maximum minutes
1	18	31 (12.6)	15–60	15	55 (18.1)	30–90
2	10	26 (16.8)	3–60	11	45 (18.6)	30–90
3	6	25 (11.6)	12–45	10	50 (17.4)	30–75

^*^Information from the forms filled out during the intervention meeting (numbers are not corresponding to the actual received meetings due to missing data)

^(**)^*OOP* oncological occupational physician

To tailor the support to the patient, patients and healthcare professionals could discuss after the first meeting whether another type of support in the intervention would be more appropriate. One patient was referred from support type B to support type A.

Thirty-two patients had a second meeting; 18 patients received (56%) the second meeting face to face (Table 2). According to protocol, the decision diagram had to be completed after the second meeting. The researchers of the project team received the checklist completed by the healthcare professional after 30 of the 32-s meetings (94%) (18 support type A/12 support type B). Accordingly, 19 (11 A/8 B) decision diagram forms were used. One patient was referred after the second meeting from support type A to support type B. Reasons for not returning the decision diagram form included the use of the wrong form during the second meeting (*N* = 1 in the support type B group), the follow-up meeting was cancelled due to the nurse taking maternity leave (*N* = 1 in the support type A group) and no follow-up meeting was needed (*N* = 3, all in the support type B group).

Seventeen patients had a third meeting of whom 11 patients received (65%) the third meeting face to face (Table 2). For 19 patients, a third meeting was not indicated, according to mutual agreement between healthcare professional and patient. The OOP needed more minutes for all their meetings compared with the nurses (Table 3).

According to protocol, at least the first two meetings should have been held, unless it was indicated that no second follow-up support was needed. Of the 38 intervention group patients, 33 (87%) had the two first meetings; three of the 38 patients did not need the second meeting.

The protocol also stated that the first meeting should be held before treatment was started. For 32% of the patients receiving support type A and 13% of those receiving support type B, this meeting was according to protocol. The timing of the other two meetings was realised according to protocol, with ≥ 80% for both types of healthcare professionals in both meetings (Table 4).

**Table 4:** Timing of the intervention meetings

	Meeting 1	Meeting 2		Meeting 3	
	Before treatment	Max. of 6 months after 1st meeting		Max. of 9 months after 1st meeting	
	*N* = 35 actual meetings	*N* = 32 actual meetings		*N* = 17 actual meetings	
	Yes, *N*/total	Yes, *N*/total	Mean in months (SD)	Yes, *N*/total	Mean in months (SD)
Nurses	6/19	18/20^*^	3 (1.5)	6/7^***^	7 (3.1)
OOP	2/16	10/12^**^	3 (2.3)	8/10^****^	7 (2.3)

^*^From the total *N* = 20

*N* = 1 no form—no information available

*N* = 1 only a second meeting—no comparison of timing with first meeting

^**^From the total *N* = 12

*N =* 1 only a second meeting—no comparison of timing with first meeting

^***^From the total *N* = 7

*N* = 1 no form—no information available

^****^From the total *N* = 10

*N* = 1 only a third meeting—no comparison of timing with first meeting

In order to conduct the intervention correctly, it was of importance to go through all the meeting items (ESM Appendices 1 and 2 for an extensive description of these items) on the checklist. The nurses had to complete 11 items in the first meeting, 14 in the second (or 12 if a third meeting was not necessary) and 10 items in the third. The OOP had to complete 13 items in the first, 15 (or 13) items in the second and 11 items in the third meeting (Table 5).

**Table 5:** The total items in the checklist which the healthcare professional should discuss in the work-related meeting, per meeting and per support type

	Meeting 1		Meeting 2, no third meeting^**^		Meeting 2		Meeting 3	
	Items^*^	Items completed	Items^*^	Items completed	Items^*^	Items completed	Items^*^	Items completed
		Mean (SD), range: min-max		Mean (SD), range: min-max		Mean (SD), range: min-max		Mean (SD), range: min-max
Support A (nurse)	11	9 (3), 11: 0–11	12	9 (2), 8: 4–12	14	9 (6), 13: 0–13	10	8 (4), 10: 0–10
Support B (OOP)	13	12 (3), 13: 0–13	13	12 (3), 6: 9–15^***^	15	13 (3), 8: 6–14	11	10 (2), 7: 4–11

^*^The total items on the checklist that the healthcare professionals should discuss in the intervention meeting

^**^No third meeting was needed; therefore, an adjusted number of items needed to be discussed

^***^OOP filled out the whole form; however, 2 questions were not needed to fill out because a third meeting was not needed afterwards

### Dose received

The process evaluation questionnaire was completed by 23 (61%) patients, six nurses who held no intervention support meetings, eight nurses who provided support and four OOPs. From the patients’ responses to the open-ended questions, we concluded that the intervention was mainly regarded as positive. From 22 responses, 11 patients experienced the intervention as useful. Fourteen (64%) patients answered the question about the general satisfaction with scores 1 and 2 (1 = very satisfied, 5 = very dissatisfied). Nevertheless, two (9%) were very dissatisfied with the intervention, stating that there had been no work-related support. The items that were discussed during the intervention meeting were scored as good by 18 of 22 patients (Table 7 in the supplementary file).

Nurses scored the training they had received before the study as either good (71%) or sufficient (29%). In response to the open questions, however, two said that they would have liked a refresher course.

The OOPs were unanimous that the time available for the first meeting was too short to provide this kind of support, while most (75%) of the nurses considered the duration to be just right (Table 8 in the supplementary file).

All nurses and OOPs stated that everything was clear before the study started and that it was easy to follow the study protocol except for one OOP, who had logistical problems.

### Fidelity

The total overall scores for fidelity were 78% for the nurses and 44% for the OOPs. The duration of the three meetings held by the OOPs was scored as not sufficient (Table 8 in the ESM), while the nurses completed all three meetings within the pre-defined average of 30 min. For most nurses and OOPs (84% and 94%, respectively), the first meetings were held face to face. Giving the first meeting before treatment was started was a challenge for both nurses and OOPs: the nurses and the OOPs managed to hold the first meeting before the start of the treatment in only 21% and 13% of cases, respectively (Table 6).

**Table 6:** Fidelity items and score-protocol adherence of the intervention performance indicators, reported for the nurses and OOPs separately

Item	Support A (nurse)	Score (%)	Score (0–1)^a^	Support B (OOP)	Score (%)	Score (0–1)
	Randomised *N* = 20			Randomised *N* = 18		
First meeting	*N* = 19 actual intervention meetings			*N* = 16 actual intervention meetings		
First meeting: before treatment started	6	32	0	2	13	0
First meeting: face to face	*N* = 16	84	1	*N* = 15	94	1
Duration 30 min	*N* = 18, mean 31 min	–	1	*N* = 15, mean 55 min	–	0
Second meeting	*N* = 20 actual intervention meetings			*N* = 12 actual intervention meetings		
Second meeting: with maximum of 6 months after first meeting (permitted by phone)	18	90	1	10	83	1
Duration 30 min	*N* = 10, mean 26 min	–	1	*N* = 11, mean 45 min	–	0
Healthcare professional filled out the decision diagram after the second meeting ^b^	11	61	0	8	67	0
Third meeting	*N* = 7 actual intervention meetings			*N* = 10 actual intervention meetings		
Third meeting (maximum of 9 months after first meeting (permitted by phone)	6	86	1	8	80	1
Duration 30 min	*N* = 6, mean 25 min	–	1	*N* = 10, mean 50 min	–	0
General item						
Healthcare professional filled out /returned the form after each meeting			1			1
First meeting	19 (19)	100		15 (16)	94	
Second meeting	18 (20)	90		12 (12)	100	
Third meeting	6 (7)	86		10 (10)	100	
Total score			78%			44%

^a^Score 0 = not sufficient and 1 = sufficient

^b^*N* = 30 checklist filled out for the second meeting (18 support A/12 support B)

## Discussion

The aim of this study was to evaluate the process of the tailored work-related support GIRONA intervention, which was received by 35 patients. Protocol adherence was scored as sufficient with a fidelity score of 78% for the nine intervention performance indicators by the oncological nurses, while the OOPs had a total fidelity score of 44%. The majority of the patients were satisfied and found the intervention useful. The healthcare professionals found the protocol easy to follow; however, they encountered some logistical hurdles, such as the timing and duration of the meetings.

### Interpretation of the findings and comparison with the literature

In our study we had an inclusion rate of 47% (process evaluation component ‘reach’), which can be considered a low percentage. Low or high inclusion rates have not be defined in the literature; however, similar ‘low’ rates were reported in other studies, all of them under 55% [23, 29–31]. A study by Tamminga et al. [23], regarding a hospital-based intervention that was targeted at patients diagnosed with cancer, reported an inclusion rate similar to that in our study. Another hospital-based study involving occupational counselling combined with physical exercise for patients diagnosed with cancer [25] reported a higher rate of participating patients (77%). Nevertheless, the study by Velthuis et al. [31], which also included an exercise programme intervention, reported a low inclusion rate of 40%. Although the same types of patients (e.g. diagnosed with cancer to be treated with curative intent) were included, the inclusion rates varied between the abovementioned studies. A possible reason for the low inclusion rate mentioned in the study by Velthuis et al. [31] is that patients declined participation before randomisation, because detailed information about the intervention was given after this step. In our study, however, we provided detailed information before randomisation, so this cannot explain our low inclusion rate. Unfortunately, we were not able to give the reasons of the non-participants. This caused by the fact that patients, by law, were not obliged to give a reason. However, these reasons would have been of added value to understand the process for those who possibly already received work-related support. Interestingly, in the study by Leensen et al. [25], the majority of the patients were diagnosed with breast cancer (*N* = 78, 84%) compared with a minor group of colorectal cancer (*N* = 8, 9%). In our study, we only included patients with gastrointestinal cancer and despite the population-based colorectal screening programme, we did not include as many as we expected. De Boer et al. [18] concluded that in this group of patients, work-related problems are already acknowledged at the moment of diagnosis. Their survey study included 333 patients who were diagnosed with GI cancer in one 7-month period at one hospital. Of those patients, 95 (28%) were employed or self-employed at the moment of diagnosis. However, in our study, we maintained other inclusion criteria, including an age of 18–63 years. In the Netherlands, the incidence rate of GI cancer in both men and women increases at the age of 64 [1], whereas we only included people up to the age of 63, which could have caused the low inclusion rate. Another reason for the low inclusion rate could be that in our study, patients were asked to participate at the moment they were diagnosed with cancer. Not all eligible patients were informed about the study by the nurses or physicians, possibly because they had limited consultation time to inform patients. Another reason could be that it is considered unethical to ask patients to participate at the moment of diagnosis. As Hubbard et al. [32] noted, the priorities in the clinic limit this kind of research. Along with Tamminga [23], and in line with the responses of the nurses in our study, we think that participation in an intervention will improve when the additional steps entailed by research requirements, e.g. information about the study, reflection period to take part in the study and contact about the other research procedures including informed consent and questionnaires, are no longer necessary.

We believe that this intervention is of value, which was also noted by healthcare professionals and patients. Thus, despite the low inclusion rate, the data derived from this study reflect the importance, feasibility and usefulness of the tailored work-related support. However, further research is needed.

Another process evaluation component to discuss is fidelity. Other intervention trials that concerned work support showed considerably higher fidelity scores than found in our study. As shown in Table 8 in the ESM, the total score for the nurses reached a score of 78%, which is somewhat comparable with the study by Tamminga [23], who assessed that on average, the nurses performed 85% of the study items. The total fidelity score for the OOPs in our study was considerably lower, namely 44%.

In the study by Leensen et al. [25], the total average fidelity score was 85%, which also included OOPs who performed some items. Conversely, in our study, of the nine (100%) fidelity score items, three were about the duration of each meeting, which per protocol was set at 30 min. All three items were scored as insufficient (33%) which is the main reason for the fidelity score for the OOPs. The nurses reported the pre-defined 30 min as acceptable: they knew their patients, because they had followed the patient journey from the start of diagnosis, so less time was needed to explore the patients’ history. In addition, the cap of 30 min for the meeting was feasible for the nurses besides their other daily activities in clinical practice. However, the OOPs needed more time to ‘get to know’ their patients (medical diagnoses, treatment and work-related outcomes/problems). Second, the longer time needed by the OOPs could be a result of the complexity of the work-related problems for which the patients were referred to them in the first place. This fidelity score was pre-established; however, on reflection, 30 min should have been the minimum number of minutes for a meeting. Although the 44% fidelity score shows that the OOPs did not follow the protocol, they did spend enough time with the patients discussing complex work-related problems, which the patients appreciated. It would therefore be appropriate to adjust the time available/needed for the meetings in the tailored intervention for the OOPs. Furthermore, research is needed to establish for which meeting (i.e. before the start of treatment or after treatment) needs more time.

The GIRONA intervention initiates tailored work-related support starting before treatment. It is known from earlier research that patients at the moment of diagnosis experience work-related problems [18]. The OOPs in our study consider it important to start early with work-related support, which is also underlined in other studies [33–36]. In our intervention, the first meeting was to be before the start of treatment, because it would inform the patients about what kind of effects the treatment could have on their work capacity, as also suggested by McGrath et al. [36]. Conversely, from the findings of this process evaluation, the question is whether it is the right moment (i.e. before treatment is started) to start work-related support. As presented in this study, the support was given before treatment in only 32% of the cases in the support type A group and 13% of the cases in the support type B group. So, time from diagnosis to start of treatment varies, but it is possible that patients start their treatment within 3 weeks. Based on the results of this process evaluation, it is a logistical challenge to provide the support before treatment. Patients’ thoughts about the timing were asked for in the process evaluation questionnaire and the majority scored this as ‘right timing’ (Table 6). However, these results must be interpreted with caution, because the patients answered the question about the timing of the meeting for the moment they actually received their work-related support. As presented, in most cases, this was after treatment. The patients were satisfied, but on the other hand, we have no information concerning patients’ satisfaction when they had the first meeting before treatment as per protocol.

### Strengths and limitations

A strength of this study is that we derived data from various sources and participants: GIRONA questionnaires with self-reported outcomes of patients, checklists completed by healthcare professionals during the intervention meetings, research logbooks completed by the research team and questionnaires on process outcomes after the intervention was performed, completed by both the patients and the healthcare professionals. This resulted in a full perspective of the process evaluation of all participating stakeholders. In addition, the study was analysed in accordance with the process evaluation key components of the Linnan and Steckler framework [21], a model that is frequently used to define and systematically report a process evaluation. We used the six most relevant key components; the seventh—implementation—was not included in our study because it is a combination score of reach, dose delivered, dose received and fidelity, which was not the main focus of our study.

A limitation of the study is that the patients who received the work-related support were spread over 15 hospitals, which in itself is not a problem if the protocol is followed. However, due to the few patients per hospital who were randomised to receive work-related support, nurses did not have many patients with whom they had to hold the work-related support meetings. Considering that, and as a nurse indicated in our study, the nurses were not familiar with providing this kind of work-related support. It is therefore possible that the meetings were not conducted as intended, resulting in the lower completion percentage of discussed items during the meetings (Table 5). At the same time, the nurses stated that they were interested in supporting patients with work-related problems and, as one indicated, a brief refresher training just before the start of the study could have supported nurses in their new role.

Another limitation of this study is the moment of the process questionnaire at the end of the follow-up. This questionnaire was sent after all participants ended their follow-up, so for some patients, this took some longer time than others. This could potentially result in ‘recall bias’, as in that some patients were not able to accurately recall the details of the intervention.

### Implications of the findings for further research and practice

Effectiveness outcome studies are well known to be executed. However, a process evaluation, just as important, helps to realise if an intervention is successful [21, 37]. An implication for future research is to include a process evaluation in the study project, to increase the value of an effectiveness study and place it in perspective.

The tailored component in our study requires improvement. Further research is needed to establish the optimal moment to provide support, and especially what is achievable in clinical practice. As we identified in this study, some patients received work-related support but did not need it, while others did not receive support but reported that they needed it. This could be linked with the timing of the work-related support given and the fact that patients do not realise that problems/work problems can arise after a certain time period, as a result of disease- and treatment-related side effects [36, 38, 39]. However, some patients simply did not need any work-related support, because proper support was provided at their workplace.

Moreover, it is necessary to define and develop the roles of healthcare providers in work-related support, as also previously mentioned by Bains et al. [40]. As a result of this study, we know that oncological nurses have an important role in identifying those who need work-related support in an early phase of the diagnosis with cancer. However, to optimise that, we first have to support oncological nurses in their work-related support role with training as a starting point, as also underlined by Stergiou-Kita [34]. To integrate the work-related support in the clinical setting, it is of importance that this kind of support achieves lasting acceptance by the ones providing it. However, intervention implementation and evaluation in general are a complex phenomenon [41, 42]. Finally, there remains a challenging hurdle in clinical practice: obtaining the time and financial support (work-related support should be recognised within the official reimbursement system) for both nurses and OOPs. For this study, ‘research’ time was allocated and a financial concession was financed from the research grant.

## Conclusion

This study, which focused on the process of the GIRONA intervention in practice, has shown that the strategy of tailored work-related support is appreciated by both patients and healthcare professionals and applicable in clinical practice. The healthcare professionals are aware that this kind of support is important for patients diagnosed with cancer to be treated with curative intent, even though not every patient is aware of the importance of work and the problems that can arise due to the diagnosis and treatment. So, first, oncological nurses have an important role in the preliminary screening of patients who are in need of tailored work-related support, and in providing such support if they are able to do so (i.e. deal with mild work-related problems); however, it is essential to train them. Second, OOPs have an important role in providing cancer patients with specialised and complex work-related support. However, both the timing and the duration of the support meetings require further research.

## Electronic supplementary material

ESM 1 (DOCX 18 kb)

ESM 2 (DOCX 66 kb)
