# Supplementary material for: Process evaluation of a tailored work-related support intervention for patients diagnosed with gastrointestinal cancer
Source: J Cancer Surviv. 2019 Nov 19;14(1):59–71. doi: 10.1007/s11764-019-00797-3 (PMC7028837; doi:10.1007/s11764-019-00797-3)
Supplement: Supplementary file 1 — (DOCX 18 kb) [file 11764_2019_797_MOESM1_ESM.docx]

Table 7. Patients experiences about the work-related support intervention (process measures: dose received)

| Process measures | N=23^*^ | | |
| --- | --- | --- | --- |
| Experience about the number of meetings ^**^  Exactly right  Not enough | 16  5 |  | |
| Experience about the duration of the meetings  Exactly right  Too short  Too long  Not applicable | First meeting  19  1  1  1 | Second meeting  12  1  -  6 | Third meeting  8  -  -  11 |
| Experience with the timing of the meetings  Exactly right  Too early  Too late  Not applicable | First meeting  20  1  1  - | Second meeting  12  -  1  8 | Third meeting  7  -  1  13 |
| Experience with the items discussed  Good  Okay  Bad | 18  2  2 |  | |
| Intervention considered as useful  Yes  Reasonably useful  Neutral  Not really  No | 11  3  5  2  1 |  | |
| General satisfaction  1 very satisfied  2  3 neutral  4  5 very dissatisfied | 7  7  5  1  2 |  | |

^*^ Due to missing items not all categories add up to N=23

^**^ In addition patients 4 patients indicated that 4, 6 or even 9 meetings would be better

Table 8. Healthcare professionals experiences about the work-related support intervention (process measures-dose received)

|  | Nurse^*^ | Nurse  intervention meetings | OOP |
| --- | --- | --- | --- |
|  | N=6 | N=8 | N=4 |
| Experience about the training  Good  Sufficient  Bad | 3  3  - | 7  1  - | NA^**^ |
| Supporting patients with work-related problems  I did not need a training for this  Yes, I had enough knowledge  No | -  5  1 | 1  7  - | NA |
| Experience about the timing of the training  Fine  Too early  Too late | 5  1  - | 7  1  - | NA |
| Missing elements in the training  Yes  No | 1  5 | 1  7 | NA |
| Experience their role to support  1 very satisfied  2  3 neutral  4  5 very dissatisfied | -  1  5  -  - | 4  -  4  -  - | 2  1  1  -  - |
| Feeling in this supporting role  Good  Neutral  Insecure  Bad | 5 | 3  2  3  - | 4 |
| Do know patients the importance of work  Yes  No | 3  3 | 2  6 | 2  2 |
| Experience about the number of meetings  Fine  Too little  Too much | NA | 7  -  1 | 3  1  - |
| Experience about the duration of the meeting (1)  Fine  Too short  Too long | NA | 6  1  1 | -  4  - |
| Experience about the duration of the meeting (2)  Fine  Too short  Too long | NA | 5  1  2 | 3  1  - |
| Experience about the duration of the meeting (3)  Fine  Too short  Too long  Not applicable | NA | 4  1  2  1 | 2  1  -  1 |
| Experience about the timing of the meeting (1)  Fine  Too early  Too late | NA | 5  2  1 | 3  -  1 |
| Experience about the timing of the meeting (2)  Fine  Too early  Too late | NA | 7  -  1 | 4  -  - |
| Experience about the timing of the meeting (3)  Fine  Too early  Too late | NA | 7  -  1 | 2  1  1 |

^*^ Nurses who had attended the training, but had no actual intervention meetings

^**^ NA= not applicable
