# Supplementary material for: Process evaluation of a tailored work-related support intervention for patients diagnosed with gastrointestinal cancer
Source: J Cancer Surviv. 2019 Nov 19;14(1):59–71. doi: 10.1007/s11764-019-00797-3 (PMC7028837; doi:10.1007/s11764-019-00797-3)
Supplement: Supplementary file 2 — (DOCX 66 kb) [file 11764_2019_797_MOESM2_ESM.docx]

***Appendix 1***

Overview discussed elements in the work-related support meetings by oncological nurse / oncological occupational physician- per intervention patient -per meeting

| Element ^A^ | Patient ID | 1 * | 2 | 3 | 4 | 5 | 6 | 7 | 8 | 9 | 10 | 11 * | 12 | 13 | 14 | 15 | 16 | 17 | 18 * | 19 * | 20 | 21 * | 22 * | 23 | 24 | 25 | 26 * | 27 | 28 | 29 | 30 * | 31 | 32 | 33 | 34 | 35 * | 36 | 37 | 38 |
| --- | --- | --- | --- | --- | --- | --- | --- | --- | --- | --- | --- | --- | --- | --- | --- | --- | --- | --- | --- | --- | --- | --- | --- | --- | --- | --- | --- | --- | --- | --- | --- | --- | --- | --- | --- | --- | --- | --- | --- |
| Obtained through | | NS | F | F | T | F | F | T | T | F | F | F | F | T | F | F | F | F | F | T | F | / | NS | F | F | F | F | F | F | F | F | F | F | F | F | F | F | F | F |
| 1.1 |  | NS | N | N | N | N | X | O | N | O | N | O | N | N | N | N | N | O | / | X | X | / | NS | O | N | O | N | O | O | O | N | N | X | N | O | N | O | X | N |
| 1.2 |  | NS | N | N | X | N | O | O | N | O | N | O | N | N | N | N | N | O | / | N | O | / | NS | O | N | O | N | O | O | O | N | N | O | N | O | N | O | O | N |
| 1.3 |  | NS | N | N | X | N | O | O | N | O | N | O | N | X | X | N | N | O | / | N | O | / | NS | O | N | O | N | O | O | O | N | N | O | N | O | N | O | O | N |
| 1.4 |  | NS | N | N | X | N | O | O | N | O | N | O | N | N | N | N | N | O | / | N | O | / | NS | O | N | O | N | O | O | O | N | N | O | X | O | X | O | O | N |
| 1.5 |  | NS | N | N | X | N | O | O | N | O | N | O | N | N | N | N | N | O | / | N | O | / | NS | O | N | O | N | O | O | O | N | N | O | N | O | N | O | O | N |
| 1.6 |  | NS | N | N | X | N | O | O | N | O | N | O | N | N | N | N | N | O | / | N | O | / | NS | O | N | O | N | O | O | O | N | N | O | N | O | N | O | O | N |
| 1.7 |  | NS | N | N | X | N | O | O | N | O | N | O | N | N | N | N | N | O | / | N | O | / | NS | O | N | O | N | O | O | O | N | N | O | N | O | N | O | O | N |
| 1.8 |  | NS | N | N | X | N | O | O | N | O | N | O | N | N | N | N | N | O | / | N | O | / | NS | O | N | O | N | O | O | O | N | N | O | N | O | N | O | O | N |
| *1.9* |  | *NS* | *-* | *-* | *-* | *-* | *O* | *O* | *-* | *O* | *-* | *O* | *-* | *-* | *-* | *-* | *-* | *O* | *-* | *-* | *O* | *-* | *NS* | *O* | *-* | *O* | *-* | *O* | *O* | *X* | *-* | *-* | *O* | *-* | *O* | *-* | *O* | *O* | *-* |
| *1.10* |  | *NS* | *-* | *-* | *-* | *-* | *O* | *O* | *-* | *O* | *-* | *O* | *-* | *-* | *-* | *-* | *-* | *O* | *-* | *-* | *O* | *-* | *NS* | *O* | *-* | *O* | *-* | *O* | *O* | *O* | *-* | *-* | *O* | *-* | *O* | *-* | *O* | *O* | *-* |
| 1.11 |  | NS | N | N | X | N | O | O | N | O | N | O | X | N | N | N | N | O | / | N | O | / | NS | O | X | O | N | O | O | O | N | N | O | N | O | N | O | O | N |
| 1.12 |  | NS | N | N | N | N | O | O | N | O | X | O | N | N | N | N | N | O | / | N | O | / | NS | O | N | O | N | O | O | O | X | N | O | N | O | N | O | O | N |
| 1.13 |  | NS | 45 | 30 | X | 20 | 30 | 40 | 15 | 60 | 45 | 75 | 30 | 15 | 20 | 30 | 20 | 60 | / | 15 | 30 | / | NS | 60 | 30 | 70 | 60 | 50 | 90 | 75 | 30 | 42 | 45 | 35 | 30 | 35 | 60 | 60 | 45 |
| Total items  Discussed | | X | 11 | 11 | 2 | 11 | 12 | 13 | 11 | 13 | 10 | 13 | 9 | 9 | 10 | 11 | 11 | 13 | 0 | 10 | 12 | 0 | X | 13 | 10 | 13 | 11 | 13 | 13 | 12 | 10 | 11 | 13 | 10 | 13 | 10 | 13 | 12 | 11 |
| Obtained through | | NS | T | T | T | F | - | T | T | F | F | NS | F | F | T | F | F | F | T | / | F | F | F | F | F | T | F | T | F | T | F | X | - | F | - | M | X | T | F |
| 2.1 |  | NS | N | N | N | N | - | O | N | O | N | NS | N | N | N | N | N | O | N | / | O | N | O | O | N | O | N | O | O | O | / | N | - | N | - | M | O | O | N |
| 2.2 |  | NS | N | N | N | N | - | O | N | O | N | NS | N | N | N | N | N | O | N | / | O | N | O | O | N | O | N | O | O | O | / | N | - | N | - | M | O | O | N |
| 2.3 |  | NS | N | N | N | N | - | O | N | O | N | NS | N | N | N | N | N | O | N | / | O | X | X | O | N | O | N | O | O | O | / | N | - | N | - | M | O | O | N |
| 2.4 |  | NS | N | X | N | N | - | O | N | O | N | NS | N | N | N | N | N | O | N | / | O | X | X | O | N | O | N | O | O | O | / | N | - | N | - | M | O | O | N |
| 2.5 |  | NS | X | X | N | N | - | O | N | O | N | NS | X | N | N | N | N | O | N | / | O | X | X | O | N | O | N | O | O | X | / | N | - | N | - | M | O | O | N |
| 2.6 |  | NS | N | X | N | N | - | O | N | O | N | NS | N | N | N | N | N | O | N | / | O | X | X | O | N | O | N | O | O | O | / | N | - | N | - | M | O | O | N |
| 2.7 |  | NS | X | X | X | N | - | O | N | O | N | NS | N | N | N | N | N | O | N | / | O | X | X | O | N | O | N | O | O | O | / | N | - | N | - | M | O | O | N |
| 2.8 |  | NS | N | X | N | N | - | O | N | O | N | NS | X | X | X | N | N | O | N | / | O | X | X | O | N | O | X | O | O | O | / | N | - | N | - | M | X | O | N |
| 2.9 |  | NS | N | X | N | N | - | O | N | O | N | NS | N | N | N | N | N | O | N | / | O | X | X | O | N | O | N | O | O | O | / | N | - | N | - | M | O | O | N |
| *2.10* |  | *NS* | *-* | *-* | *-* | *-* | *-* | *O* | *-* | *O* | *-* | *NS* | *-* | *-* | *-* | *-* | *-* | *O* | *-* | */* | *O* | *-* | *O* | *O* | *-* | *O* | *-* | *O* | *O* | *O* | */* | *-* | *-* | *-* | *-* | *M* | *O* | *O* | *-* |
| 2.11 |  | NS | - | - | - | - | - | O | - | O | N | NS | - | - | - | N | N | O | - | / | - | X | O | O | N | O | N | O | O | O | / | - | - | N | - | M | - | - | N |
| 2.12 |  | NS | N | N | N | N | - | X | N | O | N | NS | X | X | X | X | X | O | N | / | X | X | X | O | N | O | N | O | O | O | / | X | - | N | - | M | X | O | N |
| 2.13 |  | NS | - | - | - | - | - | O | - | O | N | NS | - | - | - | N | N | O | - | / | - | N | O | O | N | O | N | O | O | O | / | - | - | N | - | M | - | - | - |
| 2.14 |  | NS | X | X | X | X | - | - | X | - | - | NS | N | X | X | - | - | O | X | / | O | N | X | - | - | - | - | - | - | - | / | X | - | - | - | M | X | O | X |
| 2.15 |  | NS | X | X | X | X | - | 30 | X | 50 | 30 | NS | 20 | X | 3 | 15 | 20 | 40 | 30 | / | 30 | 10 | 60 | 60 | 45 | 40 | X | 30 | 90 | 30 | / | X | - | 60 | - | M | X | 40 | 30 |
| Total items  discussed | | X | 9 | 4 | 9 | 10 | - | 13 | 10 | 14 | 13 | X | 9 | 8 | 9 | 12 | 12 | 15 | 11 | 0 | 12 | 5 | 6 | 14 | 13 | 14 | 11 | 14 | 14 | 13 | 0 | 9 | - | 13 | - | X | 9 | 13 | 12 |
| Obtained through | | F | - | - | - | - | - | F | - | F | T | T | - | - | - | F | F | - | - | T | - | - | NS | F | F | T | / | T | F | F | F | - | - | F | - | M | - | - | - |
| 3.1 |  | O | - | - | - | - | - | O | - | O | N | O | - | - | - | N | N | - | - | N | - | - | NS | O | N | O | / | O | O | O | O | - | - | N | - | M | - | - | - |
| 3.2 |  | O | - | - | - | - | - | O | - | O | N | O | - | - | - | N | N | - | - | N | - | - | NS | O | N | O | / | O | O | O | O | - | - | N | - | M | - | - | - |
| 3.3 |  | O | - | - | - | - | - | O | - | O | N | O | - | - | - | N | N | - | - | N | - | - | NS | O | N | O | / | O | O | O | X | - | - | N | - | M | - | - | - |
| 3.4 |  | O | - | - | - | - | - | O | - | O | N | O | - | - | - | N | N | - | - | N | - | - | NS | O | N | O | / | O | O | O | X | - | - | N | - | M | - | - | - |
| 3.5 |  | O | - | - | - | - | - | O | - | O | N | O | - | - | - | N | N | - | - | N | - | - | NS | O | N | O | / | O | O | O | X | - | - | N | - | M | - | - | - |
| 3.6 |  | O | - | - | - | - | - | O | - | O | N | O | - | - | - | N | N | - | - | N | - | - | NS | O | N | O | / | O | O | O | X | - | - | N | - | M | - | - | - |
| 3.7 |  | O | - | - | - | - | - | O | - | O | N | O | - | - | - | N | N | - | - | N | - | - | NS | O | N | O | / | O | O | X | X | - | - | N | - | M | - | - | - |
| 3.8 |  | O | - | - | - | - | - | X | - | O | N | X | - | - | - | X | N | - | - | X | - | - | NS | O | X | O | / | O | O | O | X | - | - | N | - | M | - | - | - |
| *3.9* |  | *O* | *-* | *-* | *-* | *-* | *-* | *O* | *-* | *O* | *-* | *O* | *-* | *-* | *-* | *-* | *-* | *-* | *-* | *-* | *-* | *-* | *NS* | *O* | *-* | *O* | */* | *O* | *O* | *O* | *O* | *-* | *-* | *-* | *-* | *-* | *-* | *-* | *-* |
| 3.10 |  | O | - | - | - | - | - | O | - | O | N | O | - | - | - | N | N | - | - | X | - | - | NS | O | N | O | / | O | O | O | X | - | - | N | - | M | - | - | - |
| 3.11 |  | 30 | - | - | - | - | - | 60 | - | 60 | 20 | 30 | - | - | - | 20 | 20 | - | - | 12 | - | - | NS | 60 | 30 | 30 | / | 30 | 75 | 60 | 60 | - | - | 45 | - | M | - | - | - |
| Total items  discussed | | 11 | - | - | - | - | - | 10 | - | 11 | 10 | 10 | - | - | - | 9 | 10 | - | - | 8 | - | - | X | 11 | 9 | 11 | 0 | 11 | 11 | 10 | 4 | - | - | 10 | - | X | - | - | - |

^A^ see appendix 2 for the detailed description of the discussion elements per meeting

**Legend:**

Obtained through: face to face contact = F or by telephone = T

Healthcare professional: N= oncological nurse and O= oncological occupational physician

Not filled out by the healthcare professional X

Form not returned to researcher /

Not applicable -

No Show NS ^*^*(patient ID 1 (first and second meeting) / 11 (second meeting) / 22 (first and third meeting)*

Meetings cancelled due to maternity leave nurse M ^*^*(patient ID 35: follow up meetings cancelled)*

^*^ Form not returned to researcher

*18 first meeting with OOP*

*19 second meeting with oncological nurse*

*21 first meeting with oncological nurse held after treatment started = second meeting*

*26 third meeting with oncological nurse*

*30 second meeting with oncological nurse; an abstract of this meeting was provided to the researcher*

^*^ Wrong form was filled out

*21 (second meeting with nurse) form of the first meeting was used*

*22 (first meeting with OOP though second GIRONA meeting) form of first meeting was used because of No Show first meeting*

*30 (third meeting switch from nurse to oncological occupational physician) form of meeting 1 was used for the third meeting*

***Appendix 2***

Detailed description per meeting of the elements to discuss with the patient in the work-related support meetings

| 1.1  1.2  1.3  1.4  1.5  1.6  1.7  1.8  1.9  1.10  1.11  1.12  1.13 | Meeting 1  Which factors influence the limitations for the work  Are there other work-related problems  Discuss consequences due to limitations and complaints diagnosis  Is there advice for the consequences for the work  Discuss patient ‘wishes about work-stay working  Discuss whether it is feasible to continue working (during treatment)  Inform patient about the importance of work  Discuss patient ‘wishes about openness of having cancer to manager/ colleagues  *(OOP) extra– opinion OOP about work resumption*  *(OOP) extra– advice work about e.g. work content, working hours, tasks etc.*  Goals for next meeting  Planning second meeting  Duration |
| --- | --- |
| 2.1  2.2  2.3  2.4  2.5  2.6  2.7  2.8  2.9  2.10  2.11  2.12  2.13  2.14  2.15 | Meeting 2  To evaluate the work-related problems from the first meeting  Discuss new- other work/related problems compared to the first meeting  Discuss if there is contact with work environment  Discuss preparation for RTW /work already resumed?  Discuss bottlenecks resuming work  Discuss important moments within process of sick leave  Discuss elements left from the first meeting  Evaluate goal(s) from first meeting  Discuss if third meeting is necessary  *(OOP) –advice work about e.g. work content, working hours, tasks etc.*  Setting goals for third meeting  Complete decision diagram  Planning third meeting  No third meeting > evaluate work-related support  Duration |
| 3.1  3.2  3.3  3.4  3.5  3.6  3.7  3.8  3.9  3.10  3.11 | Meeting 3  To evaluate the work-related problems from the first meeting  Discuss new- other work/related problems compared to the first meeting  Discuss if there is contact with work environment  Discuss preparation for RTW /work already resumed?  Discuss bottlenecks resuming work  Discuss important moments within process of sick leave  Discuss elements left from the first/second meeting  Evaluate goal(s) from first/second meeting  *(OOP) –advice work about e.g. work content, working hours, tasks etc.*  Evaluate work-related support  Duration |
